# Knowledge, attitudes, and practices of family members of children undergoing chemoradiotherapy regarding oral mucositis

**DOI:** 10.1016/j.apjon.2025.100724

**Published:** 2025-05-15

**Authors:** Hui Gan, Kailan Chen, Li Tao, Qian Hu, Cheng Yang, Xi Xu

**Affiliations:** Department of Oncology, Wuhan Children's Hospital, Tongji Medical College, Huazhong University of Science & Technology, Wuhan, China

**Keywords:** Knowledge, Attitude, Practice, Chemoradiotherapy, Oral mucositis, Cross-sectional

## Abstract

**Objective:**

To explored the knowledge, attitudes, and practices (KAP) of family members of children receiving chemoradiotherapy concerning oral mucositis.

**Methods:**

A cross-sectional study was conducted from August 15, 2023, to May 31, 2024, in the oncology ward of Wuhan Children's Hospital of Tongji Medical College, Huazhong University of Science and Technology, using a custom-designed KAP questionnaire.

**Results:**

Of the 364 valid responses, 63.19% were from female family members. Most children (45.33%) received treatment for over six months, and 38.74% were diagnosed with oral mucositis. The participant's median (P25–P75) for knowledge, attitude, and practice scores were 6 (5, 7) (possible range: 0–7), 27 (24, 30) (possible range: 6–30), and 17 (14, 21) (possible range: 5–30), respectively. A significant 36.26% were unsure about the link between radiotherapy and oral mucositis. Only 14.56% were neutral or disagreed on the importance of special oral care. On the practical side, 69.78% of children never used dental floss and 31.59% had never visited the hospital for oral check-ups, though most participants preferred receiving information from medical professionals. Further correlation analysis revealed positive correlations between knowledge scores and attitude scores (*r* ​= ​0.270, *P ​<* ​0.001), as well as between knowledge scores and practice scores (*r* ​= ​0.164, *P* ​= ​0.002). Additionally, attitude scores were positively correlated with practice scores (*r* ​= ​0.280, *P ​<* ​0.001).

**Conclusions:**

Family members of children undergoing chemoradiotherapy have adequate knowledge and positive attitudes about oral mucositis, but this does not always lead to proactive practices. Educational interventions should enhance knowledge and encourage its application in daily caregiving to manage oral mucositis effectively.

## Introduction

Childhood cancer remains a significant public health concern worldwide, ranking as the second leading cause of death among children and adolescents and contributing substantially to the overall cancer burden.^1^^,^^2^ Approximately 400,000 children and adolescents aged 0–19 years are diagnosed with cancer annually worldwide.^3^ Notably, China accounts for a significant portion of the global incidence of childhood cancer.^4^ The primary treatments for these malignancies include surgical resection, chemoradiotherapy, and radiotherapy.^5^ Children undergoing chemoradiotherapy frequently suffer from distressing symptoms such as nausea and pain.^6^

Oral mucositis emerges as one of the most prevalent complications among pediatric cancer patients undergoing chemotherapy, radiotherapy, or hematopoietic stem cell transplantation, with an incidence rate ranging from 40% to 100%.^7^^,^^8^ Research demonstrates that while the cure rate for childhood cancer exceeds 80% in high-income countries, it remains below 30% in low- and middle-income countries.^3^ This disparity primarily stems from delayed diagnosis, inability to obtain accurate diagnosis, inaccessible therapy, and treatment abandonment. In treatment protocols, common chemotherapeutic agents such as doxorubicin, bleomycin, fluorouracil, and methotrexate are most frequently associated with the development of oral mucositis.^9^ Regarding the prevention and management of oral mucositis, studies reveal that only 29% of low-income countries report cancer medicines generally available to their populations, compared to 96% in high-income countries.^3^

Patients with OM face considerable challenges including difficulties in speaking, swallowing, and eating, which significantly impair daily functioning and overall wellbeing.^10^ Current management strategies for OM primarily focus on symptomatic relief, as effective treatments remain elusive. Expert recommendations for managing these complications include maintaining rigorous oral hygiene and using anti-inflammatory agents.^11^ The unique physiological conditions of the oral environment can impede normal healing processes, necessitating a high level of oral hygiene and the use of pharmacological agents. Furthermore, OM can lead to severe complications including intense pain, swallowing difficulties, altered taste, weight loss, and secondary infections, all of which can severely disrupt cancer treatment and dramatically reduce the patient's quality of life.^12^

The Knowledge, Attitudes, and Practices (KAP) survey serves as a pivotal diagnostic tool in health literacy research. It assesses groups' understanding, beliefs, and actions regarding specific health topics. This approach is founded on the principle that enhanced knowledge fosters positive attitudes, which subsequently shape behaviors.^13^^,^^14^ In the context of pediatric oncology, caregivers play an essential role in managing oral mucositis (OM) during chemoradiotherapy. Given the young age of children undergoing treatment, they often lack the comprehension and cooperation needed, which leads to significant distress and a sense of helplessness. Caregivers are tasked not only with routine oral care and monitoring symptom changes but also with providing emotional support as children endure the discomfort and fear associated with treatment.^15^^,^^16^

Improving caregivers' knowledge and management skills of OM is crucial. It enables them to support the children more effectively, alleviating symptoms, and ensuring the continuity and efficacy of the treatment. By empowering caregivers with knowledge and coping strategies, discomfort in children can be substantially reduced, adherence to treatment can be improved, thereby directly enhancing treatment outcomes and the quality of life for these young patients.^17^^,^^18^ Additionally, the findings of such research can assist clinicians and healthcare institutions in creating targeted educational programs and support systems. These systems, integrated as part of standard care, aim to better prepare family members for the challenges encountered during the treatment process.^19^ This study specifically explored the KAP of family members of children receiving chemoradiotherapy concerning oral mucositis, emphasizing the crucial interplay between caregiver support and clinical outcomes in pediatric cancer care.

## Methods

### Study design

This study was reported in accordance with the STROBE (Strengthening the Reporting of Observational Studies in Epidemiology) guidelines (Supplementary File 1). This cross-sectional study was conducted from August 15, 2023, to May 31, 2024, in the oncology ward of Wuhan Children's Hospital of Tongji Medical College, Huazhong University of Science and Technology.

### Participant recruitment

Participants were direct family members of pediatric cancer patients undergoing chemoradiotherapy. They were recruited by nurses actively involved in the study, with priority given to the family member most engaged in the child's care and decision-making. Inclusion criteria included being aged 18 years or older, possessing sufficient cognitive and communication abilities, and being involved in caregiving. Participants who could not provide informed consent, exhibited cognitive or communication impairments, submitted invalid questionnaires, or exceeded the established 30-min questionnaire completion time were excluded. A total of 404 questionnaires were distributed, with 364 valid responses retained after exclusions (4 participants under 18 years, 2 exceeding the time limit, and 34 with invalid responses).

### Data collection tools

The study employed a custom-designed Knowledge, Attitudes, and Practices (KAP) questionnaire (Appendix A). The development process included an extensive review of literature and clinical guidelines on oral mucositis. The questionnaire comprised four sections: demographic information, knowledge, attitude, and practice dimensions.

(1) Demographic information: Included age, gender, education, income, occupation, marital status, caregiving role, and prior experience with oral mucositis.
(2) Knowledge section: Contained seven multiple-choice questions about oral mucositis, scored as 1 point for correct answers, and 0 points for both incorrect and “uncertain” responses (score range: 0–7).
(3) Attitude section: Consisted of six Likert-scale questions ranging from 1 (very negative) to 5 (very positive), with a total score range of 6–30.
(4) Practice section: Included nine questions on oral care behaviors, combining Likert-scale items and a multiple-choice question with weighted scoring, for a total score range of 5–30.

A pilot study was conducted with 30 participants (28 valid responses retained), demonstrating good internal consistency (Cronbach's *α* ​= ​0.781 overall; 0.813, 0.860, and 0.707 for knowledge, attitude, and practice sections, respectively). The questionnaire incorporated feedback from critical care nursing experts with pediatric oncology experience to ensure clinical relevance.

### Data collection

Questionnaires were distributed both online and in paper form. Online distribution utilized a WeChat-based mini-program, while paper forms were distributed within the oncology ward. Participants submitted online questionnaires directly, and paper responses were collected by nurses and digitized by the research team. Each participant was allowed only one submission to ensure data integrity. The research team reviewed all responses for completeness, logical consistency, and validity.

### Data analysis

Data were analyzed using SPSS 27.0 (IBM, Armonk, NY, USA). Continuous variables were summarized as means and standard deviations (SD) or medians and interquartile ranges (IQR) based on their distribution, while categorical variables were presented as freuencies and percentages.

Group comparisons: Mann–Whitney U tests were applied for two-group comparisons, and Kruskal–Wallis H tests for three or more groups.

Correlation analysis: Spearman's correlation analysis was used to examine relationships among knowledge, attitude, and practice scores.

Logistic regression: Multivariate logistic regression analyzed factors influencing practice scores, dichotomized at 70% of the maximum score (21 points). Variables within univariate analysis were included. Results were reported with odds ratios (OR) and 95% confidence intervals (CI). Statistical significance was set at *P ​<* ​0.05.^20^

In the study, “*n*” denoted the sample size, and “p” was assumed to be 0.5 to ensure the calculation of the maximum sample size. The Type I error rate, “α,” was set at 0.05, leading to a “Z (1-α/2)” value of 1.64. The standard error, “δ,” was assumed to be 0.05. Anticipating an effective questionnaire response rate of 90%, the objective was to collect at least 300 completed questionnaires.

The formula for calculating the sample size was as follows:

$$N=\left(Z\;\left(1-\alpha /2\right)/\delta \right){\;}_{2}\times p\times \left(1-p\right)$$

## Results

### Basic characteristics of survey respondents

Initially, a total of 404 questionnaires were collected. Data excluded included: 1) 4 cases where respondents were under 18 years old; 2) 2 cases where response time exceeded 1800 seconds; 3) 34 cases where all answers in the knowledge section were consistently selected as “C. Uncertain”. Remaining valid data: 364 cases. Cronbach's α coefficient for questionnaire items in this study was 0.805, and for K, A, P dimensions were 0.754, 0.908, and 0.886 respectively. KMO coefficient was 0.842, indicating good internal consistency.

Among the participants, the majority (323, 88.74%) were under 50 years old, with a significant proportion being female family members (230, 63.19%) and residing in rural areas (196, 53.85%). Education levels varied, with 150 (41.21%) having attained middle school education or below. Most patients (314, 86.26%) were primarily cared for by their parents. Notably, family members who had previously experienced oral mucositis accounted for 106 (29.12%) of the sample. Among the children receiving treatment, boys represented a larger group (214, 58.79%), and the most common age range was 3–7 years (142, 39.01%). A significant number of these children (273, 75.00%) were undergoing radiotherapy or chemoradiotherapy, with treatment durations often extending beyond six months for many (165, 45.33%). Additionally, oral mucositis was diagnosed during treatment in 141 children (38.74%). The participant's median (P25–P75) knowledge, attitude, and practice scores were 6 (5, 7), 27 (24, 30), and 17 (14, 21), separately.

The study revealed significant differences in the KAP scores among participants based on various factors. Specifically, family members residing in urban areas exhibited significantly higher KAP scores compared to those in rural areas (*P* ​= ​0.002, *P* ​= ​0.022, *P* ​= ​0.002, respectively). Educational attainment also demonstrated a significant impact, with higher levels of education correlating with increased scores in knowledge (*P ​<* ​0.001), attitudes (*P* ​= ​0.013), and practices (*P* ​= ​0.003). Additionally, family members with a history of oral mucositis showed significantly higher scores in knowledge (*P* ​< ​0.001) and attitudes (*P* ​= ​0.018) compared to those without such a history, although the difference in practice scores was not statistically significant (*P* ​= ​0.056). Moreover, the type of patient's disease and the diagnosis of oral mucositis during treatment were associated with variations in KAP scores. Family members of patients with head and neck tumors had significantly higher knowledge (*P* ​= ​0.010) and practice (*P* ​= ​0.009) scores than those dealing with other diseases. Similarly, those whose patients were diagnosed with oral mucositis during treatment had higher scores in knowledge (*P* ​< ​0.001) and practices (*P* ​= ​0.017) than those whose patients were not diagnosed or were uncertain (Table 1).

**Table 1:** Basic information of patients and family members and KAP scores of patients' family members.

	*N* (%)	Knowledge, median (P25–P75)	*P*	Attitude, median (P25–P75)	*P*	Practice, median (P25–P75)	*P*
*N* ​= ​364		6 (5, 7)		27 (24, 30)		17 (14, 21)	
Age			0.346		0.901		0.610
< 50 years old	323 (88.74)	6 (5, 7)		27 (24, 30)		17 (14, 21)	
≥ 50 years old	41 (11.26)	7 (5, 7)		26 (24, 30)		16 (15, 21)	
Sex			0.186		0.060		0.785
Male	134 (36.81)	6 (5, 7)		26 (24, 29.25)		17 (14, 21)	
Female	230 (63.19)	6 (5.75, 7)		27.5 (24, 30)		17 (14, 21)	
Place of residence			0.002		0.022		0.002
Urban	168 (46.15)	7 (6, 7)		28 (24, 30)		18 (15, 21)	
Rural	196 (53.85)	6 (5, 7)		26 (24, 30)		16 (13, 20)	
Education			< 0.001		0.013		0.003
Middle school and below	150 (41.21)	6 (4, 7)		26 (24, 30)		16 (13, 19.25)	
High school/technical school	91 (25.00)	6 (6, 7)		26 (24, 29)		17 (14, 22)	
Associate degree and above	123 (33.79)	7 (6, 7)		29 (24, 30)		18 (15, 21)	
Average monthly family income (RMB)			0.060		0.536		0.010
< 5000	221 (60.71)	6 (5, 7)		26 (24, 30)		16 (14, 20.5)	
5000-10,000	115 (31.59)	7 (6, 7)		27 (24, 30)		19 (15, 21)	
> 10,000	28 (7.69)	6 (4, 7)		27 (24, 30)		16 (13.25, 19.75)	
Marital status			0.247		0.535		0.601
Married	352 (96.70)	6 (5, 7)		27 (24, 30)		17 (14, 21)	
Other	12 (3.30)	6 (5, 6.75)		26 (23.25, 29.5)		16.5 (13.5, 19)	
Employment status			0.003		0.831		0.086
Employed	127 (34.89)	7 (6, 7)		27 (24, 30)		18 (15, 21)	
Other	237 (65.11)	6 (5, 7)		26 (24, 30)		16 (14, 21)	
Does anyone in the family work in a medical-related profession?			0.069		0.262		0.160
Yes	68 (18.68)	7 (6, 7)		28 (24, 30)		18 (15, 21)	
No	296 (81.32)	6 (5, 7)		26.5 (24, 30)		18 (14, 21)	
Who cares for the patient during treatment?			0.744		0.867		0.956
Parents	314 (86.26)	6 (5, 7)		27 (24, 30)		17 (14, 21)	
Other	50 (13.74)	6 (5, 7)		26 (24, 30)		17.5 (14, 21)	
Has anyone in the patient's family ever had oral mucositis?			< 0.001		0.018		0.056
Yes	106 (29.12)	7 (6, 7)		28.5 (24, 30)		18 (15, 21)	
No	258 (70.88)	6 (5, 7)		26 (24, 29)		17 (14, 21)	
**Patient information**							
**Patient's sex**			0.506		0.045		0.276
Male	214 (58.79)	6 (5, 7)		27.5 (24, 30)		17 (14, 25)	
Female	150 (41.21)	6 (5, 7)		26 (24, 30)		17 (14, 24)	
**Patient's age**			0.218		0.150		< 0.001
Below 3 years old	88 (24.18)	6 (5.25, 7)		27 (24, 29)		14 (11, 19)	
3–7 years old	142 (39.01)	6 (6, 7)		27.5 (24, 30)		18 (15, 21)	
Above 7 years old	134 (36.81)	6 (5, 7)		26 (24, 30)		18 (15, 21)	
**Type of disease**			0.010		0.088		0.009
Head and neck tumor	91 (25.00)	7 (6, 7)		29 (24, 30)		18 (16, 22)	
Other	273 (75.00)	6 (5, 7)		26.5 (24, 29)		17 (14, 20)	
**When was the patient diagnosed?**			0.171		0.896		0.353
Within the past year	200 (54.95)	6 (5, 7)		27 (24, 30)		17 (14, 21)	
Over a year ago	164 (45.05)	6 (5.25, 7)		26.5 (24, 30)		18 (15, 20.75)	
**Treatment method**			0.387		0.571		0.633
Radiation or chemoradiotherapy	273 (75.00)	6 (5, 7)		27 (24, 30)		17 (14, 21)	
Radiation and chemoradiotherapy	91 (25.00)	7 (5, 7)		27 (24, 30)		17 (14, 20)	
**Duration of radiation and chemoradiotherapy treatment**			0.004		0.226		0.082
Less than one month	32 (8.79)	6 (4.25, 7)		25.5 (24, 29)		16 (12, 19)	
More than one month but less than six months	87 (23.90)	6 (4, 7)		27 (24, 30)		17 (14, 20)	
More than six months but less than one year	165 (45.33)	6 (6, 7)		27 (24, 30)		17 (15, 21)	
Over one year	80 (21.98)	6 (6, 7)		27.5 (24, 30)		18 (15, 21.75)	
Has oral mucositis been diagnosed during treatment?			< 0.001		0.125		0.017
Yes	141 (38.74)	7 (6, 7)		28 (24, 30)		18 (15, 21)	
No/not sure	223 (61.26)	6 (5, 7)		26 (24, 29)		16 (13, 21)	

KAP, The Knowledge, Attitudes, and Practices.

### Distribution of options in the knowledge, attitude, and practice

The analysis of the knowledge dimension revealed clinically significant gaps, particularly concerning the association of radiotherapy, specifically targeting the head and neck region, with the onset of oral mucositis. Notably, 36.26% of respondents were uncertain about this link. Additionally, 14.56% of the respondents were not aware that chemoradiotherapy could lead to the development of oral mucositis. Furthermore, 19.23% of the participants could not correctly identify the symptoms of oral mucositis, and there are still some patients (14.01%) who are not clear on how to maintain oral hygiene to prevent oral mucositis (Table 2).

**Table 2:** Knowledge dimension of the participants.

	*N* (%)		
	True	False	Not sure
1. Oral mucositis refers to erythema or ulcers on the oral mucosa	**284 (78.02)**	10 (2.75)	70 (19.23)
2. Chemoradiotherapy can lead to the occurrence of oral mucositis	**309 (84.89)**	2 (0.55)	53 (14.56)
3. Radiotherapy can lead to the occurrence of oral mucositis	**215 (59.07)**	17 (4.67)	132 (36.26)
4. Oral mucositis can cause pain, difficulty eating, and taste disturbances	**336 (91.31)**	4 (1.10)	24 (6.59)
5. Improper oral hygiene is a common factor leading to oral mucositis	**319 (87.64)**	5 (1.37)	40 (10.99)
6. Knowing how to properly clean the mouth and maintain oral health helps prevent oral mucositis	**312 (85.71)**	1 (0.27)	51 (14.01)
7. Oral mucositis can be alleviated through medication	**329 (90.38)**	0	35 (9.62)

Bolded options are correct.

The analysis of participants' attitudes toward oral mucositis revealed predominantly positive responses, indicating a broad recognition of its clinical significance. However, some replies emphasized areas where further educational efforts might be beneficial. Specifically, while the majority of people believe that oral mucositis can have a negative impact on the quality of life for children, 9.62% of individuals remain neutral (A1). Moreover, 14.56% do not think special attention to oral mucosal health is necessary (6.87 strongly agree, 3.02 agree, 4.67 neutral) (A2) (Table 3).

**Table 3:** Attitude dimension of the participants.

	*N* (%)				
	Strongly agree	Agree	Neutral	Disagree	Strongly disagree
1. Do you believe that oral mucositis has a significant negative impact on the child's quality of life? P	201 (55.22)	123 (33.79)	35 (9.62)	3 (0.82)	2 (0.55)
2. Do you think that the oral mucosa does not need special attention? N	25 (6.87)	11 (3.02)	17 (4.67)	148 (40.66)	163 (44.78)
3. Do you believe that maintaining oral hygiene can prevent oral mucositis? P	192 (52.75)	144 (39.56)	25 (6.87)	2 (0.55)	1 (0.27)
4. Do you believe that it is important to understand the harm and preventive measures of oral mucositis? P	208 (57.14)	135 (37.09)	19 (5.22)	1 (0.27)	1 (0.27)
5. Do you believe that oral mucositis should be actively treated and managed in its early stages? P	213 (58.52)	130 (35.71)	19 (5.22)	1 (0.27)	1 (0.27)
6. Do you believe that more attention should be given to the prevention and treatment of oral mucositis? P	207 (56.87)	138 (37.91)	17 (4.67)	1 (0.27)	1 (0.27)

P is the positive score assigned to the positive question; N is the negative score assigned to the negative question.

The practice dimension of participants related to oral health care in affected children highlights several areas of concern, as well as opportunities for improved patient education and support. Notably, a significant proportion of the participants reported infrequent engagement in key oral health practices. Specifically, 69.78% of children reportedly never used dental floss, and 31.59% had never been taken to the hospital for oral health check-ups. Furthermore, more than half of the participants (54.95%) did not encourage their children to seek timely medical or dental care. These findings indicate a substantial lack of engagement in recommended oral health care practices. Conversely, the data also reveal a strong preference among participants for receiving information about oral mucositis from medical professionals, with 89.29% expressing a preference for information provided by doctors or hospitals (Table 4).

**Table 4:** Practice dimension of the participants.

	*N* (%)				
	Always	Often	Sometimes	Occasionally	Never
For the following behaviors, how often does the child perform them:					
1. **Brushing teeth in the morning and evening**	179 (49.18)	86 (23.63)	51 (14.01)	32 (8.79)	16 (4.40)
2. **Rinsing mouth after meals**	87 (23.90)	63 (17.31)	95 (26.10)	66 (18.13)	53 (14.56)
3. **Using dental floss**	30 (8.24)	9 (2.47)	26 (7.14)	45 (12.36)	254 (69.78)
4. **Avoiding irritating foods or drinks**	128 (35.16)	88 (24.18)	46 (12.64)	63 (17.31)	39 (10.71)
5. **Regularly visiting the hospital for oral health check-ups**	45 (12.36)	25 (6.87)	50 (13.74)	129 (35.44)	115 (31.59)
	Yes			No	
6. **Do you pay attention to the child's oral health during radiotherapy or chemoradiotherapy?**	335 (92.03)			29 (7.97)	
8. **Do you need more information on the prevention and treatment of oral mucositis?**	311 (85.44)			53 (14.56)	
7. **What measures have you taken to maintain the child's oral health?**					
a. Educating the child on oral hygiene habits such as brushing and rinsing	341 (93.68)			23 (6.32)	
b. Consulting with doctors or dentists for oral care advice	240 (65.93)			124 (34.07)	
c. Providing soft foods or a diet suitable for oral mucositis patients	248 (68.13)			116 (31.87)	
d. Encouraging the child to seek timely medical or dental care	164 (45.05)			200 (54.95)	
e. Other	4 (1.10)			360 (98.90)	
f. Have not taken any measures	14 (3.85)			350 (96.15)	
9. **From which of the following sources would you prefer to obtain information on oral mucositis?**					
a. Information provided by doctors or hospitals	325 (89.29)			39 (10.71)	
b. Online searches	187 (51.37)			177 (48.63)	
c. Community health education activities	145 (39.84)			219 (60.16)	
d. Advice from oral specialists	225 (61.81)			139 (38.19)	
e. Other	363 (99.73)			1 (0.27)	
f. Do not need more information	9 (2.47)			355 (97.53)	

### Correlation analysis of knowledge, attitude, and practice

Further correlation analysis revealed positive correlations between knowledge scores and attitude scores (*r* ​= ​0.270, *P* ​< ​0.001), as well as between knowledge scores and practice scores (*r* ​= ​0.164, *P* ​= ​0.002). Additionally, attitude scores were positively correlated with practice scores (*r* ​= ​0.280, *P* ​< ​0.001) (Table 5). Multivariate logistic regression showed that higher attitude scores were protective factors (OR ​= ​1.094, 95% CI [1.006–1.190], *P* ​= ​0.036) for more independently positive practice (Table 6).

**Table 5:** Correlation analysis of KAP scores.

	Knowledge	Attitude	Practice
Knowledge	1		
Attitude	0.270 (*P* ​< ​0.001)	1	
Practice	0.164 (*P* ​= ​0.002)	0.280 (*P* ​< ​0.001)	1

KAP, The Knowledge, Attitudes, and Practices.

**Table 6:** Factors of practice based univariate and multivariate logistic regression.

Practice*	Univariate logistic regression		Multivariate logistic regression	
	OR (95% CI)	*P*	OR (95% CI)	*P*
Knowledge dimension	1.146 (0.976–1.347)	0.097	1.045 (0.877–1.245)	0.624
Attitude dimension	1.111 (1.026–1.202)	< 0.001	1.094 (1.006–1.190)	0.036
Age (years)				
< 50	0.721 (0.356–1.458)	0.362		
≥ 50	Ref			
Sex				
Male	1.025 (0.630–1.666)	0.922		
Female	Ref			
Place of residence				
Urban	1.304 (0.815–2.088)	0.268		
Rural	Ref			
Education				
Middle school and below	0.738 (0.425–1.283)	0.282		
High school/technical school	1.104 (0.607–2.010)	0.745		
Associate degree and above	Ref			
Average monthly family income (RMB)				
< 5000	Ref		Ref	
5000-10,000	1.320 (0.800–2.179)	0.277	1.281 (0.760–2.160)	0.352
> 10,000	0.503 (0.167–1.514)	0.221	0.492 (0.158–1.530)	0.221
Marital status				
Married	1.769 (0.381–8.225)	0.467		
Other	Ref			
Employment status				
Employed	1.013 (0.619–1.657)	0.959		
Other	Ref			
Does anyone in the family work in a medical-related profession?				
Yes	1.143 (0.633–2.063)	0.976		
No	Ref			
Who cares for the patient during treatment?				
Parents	0.989 (0.501–1.954)			
Other	Ref			
Has anyone in the patient's family ever had oral mucositis?				
Yes	0.974 (0.580–1.636)	0.922		
No	Ref			
Patient's gender				
Male	1.177 (0.728–1.904)	0.506		
Female	Ref			
Patient's age				
Below 3 years old	Ref		Ref	
3–7 years old	1.526 (0.799–2.913)	0.2	1.290 (0.659–2.526)	0.457
Above 7 years old	1.715 (0.897–3.275)	0.103	1.463 (0.744–2.880)	0.270
Type of disease				
Head and neck tumor	1.497 (0.889–2.521)	0.13	1.454 (0.835–2.533)	0.186
Other	Ref		Ref	
When was the patient diagnosed?				
Within the past year	1.082 (0.674–1.735)	0.745		
Over a year ago	Ref			
Treatment method				
Radiation or chemoradiotherapy	1.435 (0.811–2.541)	0.215	1.816 (0.982–3.360)	0.057
Radiation and chemoradiotherapy	Ref		Ref	
Duration of radiation and chemoradiotherapy treatment				
Less than one month	Ref		Ref	
More than one month but less than six months	1.612 (0.549–4.733)	0.385	1.444 (0.482–4.330)	0.512
More than six months but less than one year	1.903 (0.689–5.255)	0.214	1.721 (0.605–4.892)	0.309
Over one year	2.600 (0.898–7.525)	0.078	2.335 (0.769–7.088)	0.134
Has oral mucositis been diagnosed during treatment?				
Yes	0.975 (0.602–1.580)	0.919		
No/not sure	Ref			

^∗^: Defined as a score ≥ 21 (70% of total) indicating good practice (*n* = 94); < 21 indicating poor practice (*n* = 270).

## Discussion

Family members of children undergoing chemoradiotherapy generally possess adequate knowledge and positive attitudes towards oral mucositis, yet their practices remain less active than expected. It is crucial to develop targeted educational interventions that not only enhance knowledge and attitudes but also actively promote best practice behaviors among family caregivers to improve the management of oral mucositis in children undergoing chemoradiotherapy.

The findings of this study indicate that family members of children undergoing chemoradiotherapy generally possess adequate knowledge and hold positive attitudes towards oral mucositis. However, their practices regarding oral mucositis management are less active than might be expected given their level of knowledge and attitudes. This gap between knowledge and practice is a common phenomenon observed in various healthcare contexts and has been noted as a significant barrier to optimal disease management and patient compliance.^21^^,^^22^

The relationship among KAP in our study was further elucidated through correlation analyses and multivariate logistic regression. Significant positive correlations were identified between knowledge and attitudes (*r* ​= ​0.270, *P* ​< ​0.001), knowledge and practices (*r* ​= ​0.164, *P* ​= ​0.002), and between attitudes and practices (*r* ​= ​0.280, *P* ​< ​0.001). These correlations suggest that improvements in one domain can potentially influence the other areas positively. Multivariate logistic regression supported these findings, particularly highlighting the attitude dimension as an independent predictor of proactive practices (OR ​= ​1.094, *P* ​= ​0.036). This aligns with literature that emphasizes the pivotal role of attitudes in translating knowledge into practice.^23^^,^^24^

Education and place of residence, notably, demonstrated marked disparities. Individuals with higher educational levels or those residing in urban areas exhibited superior KAP scores. This can be attributed to the broader access to healthcare information and resources typically available in urban settings and among more educated populations.^25^ The educational disparity underscores the role of health literacy in disease management, suggesting that individuals with more education are better equipped to assimilate and apply health-related information.

A critical finding from our study is the significantly poorer practice scores among families caring for children under three years old. This age group presents unique communication and care challenges, as these younger children may have more difficulty expressing their symptoms and needs, complicating effective management of conditions like oral mucositis. This difficulty in communication might hinder the implementation of best practices in oral care.^17^^,^^19^^,^^26^

The absence of significant differences in KAP scores related to age and gender suggests that in the context of pediatric oncology, where a child's severe health condition like cancer is the central concern, these demographic factors might play a less pivotal role compared to other health contexts. Typically, variations in health behaviors and perceptions can be prominent across different ages and genders. However, when it comes to caring for a child undergoing chemoradiotherapy, caregivers—regardless of their age or gender—are likely to demonstrate a uniformly high level of commitment and attentiveness.^27^^,^^28^ This scenario underscores a universal caregiver response that transcends typical demographic divisions, highlighting a collective prioritization of the child's health above other factors. Such insights suggest that interventions to improve KAP among caregivers might be more effective if they are designed recognizing this inherent high level of concern, rather than tailored specifically by age or gender.

A notable gap in understanding remains, as 36% of family members are unaware that radiotherapy can cause oral mucositis, and only 59% correctly identified its role. This highlights the need for targeted educational efforts to address these misconceptions. It is important to note, however, that oral mucositis is primarily associated with radiotherapy targeting the head and neck region, as the oral mucosa is directly exposed to radiation. Radiotherapy involving other body regions typically has a lower risk of inducing oral mucositis. This gap is significant considering the critical importance of managing all potential causes in a chemoradiotherapy context. Similar challenges in knowledge levels about the causes of complications during cancer treatments have been shown in other studies.^29^^,^^30^ To improve this, targeted educational interventions should be implemented, including detailed educational sessions that specifically address the comprehensive causes of oral mucositis, utilizing visual aids and interactive content to enhance understanding. Adapting these sessions to varying educational levels ensures accessibility and comprehension.^31^^,^^32^

Attitudes toward the impact and management of oral mucositis are predominantly positive, with a strong majority recognizing its negative impact on quality of life and the necessity of early management. However, a smaller percentage, though significant, remains neutral or unsure about the importance of preventive measures. This ambivalence could hinder proactive health behaviors, as even a small degree of uncertainty could lead to delays in seeking care or implementing preventive measures.^33^^,^^34^ To counteract this, it is essential to foster a more proactive attitude through regular counseling sessions and testimonials from other families who have successfully managed oral mucositis. Incorporating feedback from families who have experienced these benefits firsthand can help emphasize the practical advantages of early and active management.^35^^,^^36^

The practice dimension reveals a critical area for improvement, particularly in the regularity of dental care routines such as using dental floss, which is remarkably low. Similarly, regular hospital visits for oral health during treatments are not adequately adhered to by a substantial portion of respondents. Enhancing these practices requires practical, hands-on training sessions for caregivers on how to effectively conduct oral hygiene practices at home. Additionally, setting up reminder systems, possibly through mobile health apps, could improve adherence to regular dental check-ups and daily oral care routines. Providing personalized guidance based on specific patient needs and family circumstances, especially focusing on those with lower educational backgrounds and residing in rural areas, could further tailor interventions for better practical outcomes.^37^^,^^38^

### Implications for nursing practice and research

This study highlights crucial implications for pediatric oncology nursing. Nurses should actively assess not only family members' knowledge and attitudes regarding oral mucositis but specifically their daily care practices, recognizing the identified gap. Educational interventions must move beyond general information to address specific deficits, such as the radiotherapy link, symptom identification, and practical skills like flossing and regular check-ups, utilizing the preferred medical professional source. Tailoring education based on caregiver education level, residence, and child's age (especially under 3) is vital. Future research should focus on developing and evaluating the effectiveness of targeted, practical interventions, possibly incorporating mobile health tools, to bridge the knowledge-practice gap. Longitudinal studies are needed to explore causality and the impact of interventions on clinical outcomes and caregiver burden, particularly within identified high-risk subgroups.

### Limitations

This study has several limitations that should be considered. First, its cross-sectional design limits the ability to establish causality between the knowledge, attitudes, and practices of family members and the management outcomes of oral mucositis. First, the classification of disease types into “head and neck tumors” and “other” was based on the significantly higher risk of oral mucositis in patients with head and neck malignancies. While this approach simplified data processing, it may have overlooked variations in oral mucositis risk among other tumor types. For instance, certain tumor types (e.g., hematologic malignancies) may also carry a high risk of oral mucositis, while the risk in other solid tumors may be lower. Future studies should adopt a more detailed disease classification to better capture the impact of different tumor types on oral mucositis risk. Third, the study utilized a self-designed questionnaire, which is not properly standardized psychometrically. While the questionnaire was tailored to the specific context, this limitation may impact its reliability and broader applicability. Forth, this study included family members of all children undergoing chemoradiotherapy without distinguishing between chemotherapy regimens, radiotherapy sites, or other treatment-related factors to ensure inclusivity. However, this broad inclusion approach may limit the applicability of the findings, as the risk of oral mucositis varies significantly with different chemotherapy agents (e.g., methotrexate, fluorouracil) and radiotherapy sites (e.g., head and neck radiotherapy). Future research should incorporate more detailed analyses of treatment regimens, such as differentiating high-risk chemotherapy agents and radiotherapy sites, to provide more targeted conclusions. Additionally, by including all direct family members rather than restricting the sample to primary caregivers, this study provides a broader understanding of the family environment's impact. However, this approach introduces sample heterogeneity, as the emotional experiences, stress levels, and coping strategies of direct family members may differ from those of primary caregivers. Future research could address this limitation by focusing specifically on the experiences of primary caregivers to complement the findings of this study.

## Conclusions

In conclusion, family members of children undergoing chemoradiotherapy demonstrated generally adequate knowledge and positive attitudes towards oral mucositis, yet their practices remained suboptimal. It is imperative to develop targeted educational programs that not only enhance knowledge and attitudes but also actively promote best practices in the management of oral mucositis among these family members.

## CRediT authorship contribution statement

**Hui Gan** and **Kailan Che**n carried out the studies, participated in collecting data, and drafted the manuscript. **Li Ta**o and **Chen Yang** performed the statistical analysis and participated in its design. **Qian Hu** and **Xi Xu** participated in acquisition, analysis, or interpretation of data and draft the manuscript. All authors read and approved the final manuscript.

## Ethics statement

This study was approved by the Ethics Committee of Wuhan Children's Hospital (Approval No. 2023R046-E01) and was conducted in accordance with the 1964 Helsinki Declaration and its later amendments or comparable ethical standards. All participants provided written informed consent.

## Data availability statement

The authors confirm that the data supporting the findings of this study are available within the article and its supplementary materials.

## Declaration of generative AI and AI-assisted technologies in the writing process

No AI tools/services were used during the preparation of this work.

## Funding

This study received no external funding.

## Declaration of competing interest

The authors declare no conflict of interest.
