# Supplementary material for: Knowledge, attitudes, and practices of family members of children undergoing chemoradiotherapy regarding oral mucositis
Source: Asia Pac J Oncol Nurs. 2025 May 15;12:100724. doi: 10.1016/j.apjon.2025.100724 (PMC12212175; doi:10.1016/j.apjon.2025.100724)
Supplement: Multimedia component 2 [file mmc2.docx]

Appendix A

Knowledge, Attitudes, and Practices (KAP) of Family Members of Children Undergoing Radiotherapy and Chemotherapy Regarding Oral Mucositis

Questionnaire Number:

| Dear Friend,  We are researchers from **Hospital, and we sincerely invite you to participate in our research study. This study aims to understand the knowledge, attitudes, and practices of family members of children undergoing radiotherapy and chemotherapy regarding oral mucositis. The findings will help in formulating scientific early intervention strategies, potentially aiding more individuals and improving patient health in the future. Your participation in this study is voluntary. If you agree to participate, please refer to the following instructions:  1. Please complete the questionnaire. There are no right or wrong answers; simply fill in based on your actual situation. If you encounter any problems while answering, feel free to ask us. Once completed, please submit the questionnaire promptly.  2. This study is a simple questionnaire survey and will not harm your physical or psychological health. However, it will involve some privacy-related questions, such as your gender and age. We will keep your information strictly confidential and will not disclose it. Please feel assured to fill it out.  3. As a participant, you can always access information related to this study and its progress. If you decide to withdraw from the study, please inform us, and your data will not be included in the research results.  Finally, we sincerely thank you for taking the time to support our scientific research!  □ I have been informed and agree to the use of the collected data for scientific research.  Informed Consent Signature:  Participation Date：      year      month      day |
| --- |

| **Part 1 Basic Information** | |
| --- | --- |
| 1. **Your age： years old** | |
| 1. **Your gender：** | a. male  b. female |
| 1. **Place of residence：** | a. urban  b.rural |
| 1. **Level of education：** | a.primary school or below  b.middle school  c.high school/technical school  d.associate degree  e.bachelor’s degree  f.master’s degree or above |
| 1. **Your family's average monthly income (in RMB):** | a.<5000  b.5000-10000  c.10001-20000  d.>20000 |
| **6. marital status：** | a.single  b.married  c.divorced  d.widowed |
| **7. current employment status：** | a.employed  b.unemployed  c.retired  d.self-employed  e.housewife  f.other |
| **8. Is there anyone in your family who works in a medical-related field?** | a.yes b.no |
| **9. Who takes care of the patient during treatment?** | a.parents  b.grandparents  c.nanny |
| **10.** **Has anyone in the patient's family ever had oral mucositis?** | a.yes b.no |

| Part 2 **Basic Information of the Patient** | | |
| --- | --- | --- |
| **1.patient’s gender：** | a.male b. female | |
| **2.patient’s age：** | | a.less than 1 year old b.1-3 years old c. 3-7 years old d. more than 7 years old |
| **3. type of disease** | a. Head and neck tumor  b. Other  c. not sure | |
| **4. When was the child diagnosed?** | a. within three months  b.within six months  c.within one year  d.over a year age | |
| **5. type of treatment** | a.radiotherapy  b.chemotherapy  c.both radiotherapy and chemotherapy | |
| **6.** **Duration of radiotherapy and/or chemotherapy:** | a. Less than one month  b. More than one month but less than six months  c. More than six months but less than one year  d. More than one year | |
| **7.** **Has the patient been diagnosed with oral mucositis during treatment?** | a.yes b.no c.not sure | |

| **Part 3 Knowledge About Oral Mucositis in Children** | | | |
| --- | --- | --- | --- |
| 1. **Oral mucositis refers to erythema or ulcers on the oral mucosa:** | a.true | b.false | c.not sure |
| 1. **Chemotherapy can lead to the occurrence of oral mucositis:** | a.true | b.false | c.not sure |
| 1. **Radiotherapy can lead to the occurrence of oral mucositis:** | a.true | b.false | c.not sure |
| 1. **Oral mucositis can cause pain, difficulty eating, and taste disturbances:** | a.true | b.false | c.not sure |
| 1. **Improper oral hygiene is a common factor leading to oral mucositis:** | a.true | b.false | c.not sure |
| 1. **Knowing how to properly clean the mouth and maintain oral health helps prevent oral mucositis:** | a.true | b.false | c.not sure |
| 1. **Oral mucositis can be alleviated through medication** | a.true | b.false | c.not sure |

| **Part 4 Attitudes on Oral Mucositis** | | | | | |
| --- | --- | --- | --- | --- | --- |
| 1. **Do you believe that oral mucositis has a significant negative impact on the child's quality of life?** | a.strongly agree | b.agree | c. neutral | d.disagree | e.strongly disagree |
| 1. **Do you believe that maintaining oral hygiene can prevent oral mucositis?** | a.strongly agree | b.agree | c. neutral | d.disagree | e.strongly disagree |
| 1. **Do you believe that it is important to understand the harm and preventive measures of oral mucositis?** | a.strongly agree | b.agree | c. neutral | d.disagree | e.strongly disagree |
| 1. **Do you believe that oral mucositis should be actively treated and managed in its early stages?** | a.strongly agree | b.agree | c. neutral | d.disagree | e.strongly disagree |
| 1. **Do you believe that more attention should be given to the prevention and treatment of oral mucositis?** | a.strongly agree | b.agree | c. neutral | d.disagree | e.strongly disagree |

**Part 5 Practices for Preventing Oral Mucositis**

| For the following behaviors, how often does the child perform them: | | | | | | | | | |
| --- | --- | --- | --- | --- | --- | --- | --- | --- | --- |
| **1.** **Brushing teeth in the morning and evening:** | | | a.always | b.often | | c.sometimes | | d.occasionally | e.never |
| **2.** **Rinsing mouth after meals:** | | | a.always | b.often | | c.sometimes | | d.occasionally | e.never |
| **3.** **Using dental floss:** | | | a.always | b.often | | c.sometimes | | d.occasionally | e.never |
| **4.** **Avoiding irritating foods or drinks:** | | | a.always | b.often | | c.sometimes | | d.occasionally | e.never |
| **5.** **Regularly visiting the hospital for oral health check-ups:** | | | a.always | b.often | | c.sometimes | | d.occasionally | e.never |
| **6.** **Do you pay attention to the child's oral health during radiotherapy or chemotherapy?** | | | a.yes | | | | b.no | | |
| **7.** **What measures have you taken to maintain the child's oral health? (multiple choices)** | | | | | | | | | |
| a. Educating the child on oral hygiene habits such as brushing and rinsing | | | | | | | | | |
| b. Consulting with doctors or dentists for oral care advice | | | | | | | | | |
| c. Providing soft foods or a diet suitable for oral mucositis patients | | | | | | | | | |
| d. Encouraging the child to seek timely medical or dental care | | | | | | | | | |
| e. Other (please specify)____________ | | | | | | | | | |
| f. Have not taken any measures | | | | | | | | | |
| **8. Do you need more information on the prevention and treatment of oral mucositis?** |  | **a.yes** | | | **b.no** | | | | |
| **9.** **From which of the following sources would you prefer to obtain information on oral mucositis? (multiple choices)** | | | | | | | | | |
| a. Information provided by doctors or hospitals | | | | | | | | | |
| b. Online searches | | | | | | | | | |
| c. Community health education activities | | | | | | | | | |
| d. Advice from oral specialists | | | | | | | | | |
| e. Other (please specify) _____________ | | | | | | | | | |
| f. Do not need more information | | | | | | | | | |
